# Investigation and analysis of high risk factors of falls in hospitalised patients after vascular surgery

**DOI:** 10.1002/nop2.1987

**Published:** 2023-08-23

**Authors:** Yuan Bai, Xiao‐Hong Zhang, Fang He, Zhuo‐Xia Li, Min Li, Ying Yu

**Affiliations:** ^1^ General Surgery Department, Shanxi Bethune Hospital Taiyuan China; ^2^ Tongji Hospital, Tongji Medical College, Huazhong University of Science and Technology Wuhan China; ^3^ Vascular Surgery Department Shanxi Bethune Hospital Taiyuan China; ^4^ Nursing Department Shanxi Bethune Hospital Taiyuan China; ^5^ Breast Surgery Department Shanxi Bethune Hospital Taiyuan China

**Keywords:** countermeasures, fall, high risk factors, hospitalised patients, vascular surgery

## Abstract

**Aim:**

To analyse the risk factors for falls in vascular patients and methods to mitigate fall risk in hospitalised patients receiving vascular surgery.

**Design:**

This study is a multicentre, retrospective study.

**Methods:**

A total of 112 inpatients that underwent vascular surgery in five hospitals in Shanxi Province from April 2018 to April 2022 were selected. They were divided into two groups according to whether they had fallen or not; 56 patients who fell were taken as the observation group and 56 patients who did not fall were taken as the control group. The risk factors of falls were analysed by univariate and logistic regression.

**Results:**

There was no significant difference between the observation and the control groups in male patients and the incidence of falls without family members. In the observation group, the percentage of patients aged ≥65 years old, with a history of falls and/or fractures, long‐term medications and a history of osteoporosis was higher than in the control group and showed a statistically significant difference. Multivariate logistic regression analysis showed that advanced age, a history of falls and fractures, long‐term medications and a history of osteoporosis were independent risk factors for falls, and the differences were statistically significant.

**Conclusion:**

Older age, a history of falls and/or fractures, continuous medication for more than 3 months and a history of osteoporosis are the risk factors for falls in hospitalised patients undergoing vascular surgery.

## 1 INTRODUCTION

Vascular surgical diseases involve malformation, venous thrombosis, arteritis, arteriosclerotic occlusion, aortic aneurysm, valvular heart disease, congenital heart disease and other diseases. Patients generally need to receive surgical treatment. Due to the influence of factors such as pain, self‐care ability is reduced, limb movement is inconvenient and risk events such as falls can easily occur (Zhao et al., 2021). The incidence of falls has been increasing in recent years. Among seniors worldwide, approximately 30% of individuals aged 65 and older experience falls each year. Despite collaborative endeavours by researchers and clinicians to comprehend, evaluate and address the risks and causes, the impact of falls on this demographic remains significant. Apart from the personal distress caused, falls and fall‐related injuries present a substantial healthcare challenge due to their association with subsequent illness, impairment, hospitalisation, institutionalisation and mortality (Montero‐Odasso et al., 2022; Morris et al., 2021). Falls can also cause fractures, soft tissue damage and other adverse events, exacerbating the original disease and the psychological burden on patients, resulting in limited mobility and imposing a heavy burden on society and families (Chen et al., 2021). Based on this evidence, the present study aimed to further analyse the risk factors and response measures of fall incidents in hospitalised patients following vascular surgery.

## 2 METHODS

### 2.1 Study population

This research is a multicentre, retrospective study that was conducted at five hospitals in Shanxi Province from April 2018 to April 2022. Inclusion criteria: (1) hospitalised patients who underwent vascular surgery; (2) no preoperative risk of falls (Morse Falls Assessment Scale <45); (3) aged 30–80; (4) good coordination and compliance. Exclusion criteria: (1) lactating, pregnant women; (2) bed‐bound, bilateral amputation; (3) patients who experienced a preoperative fall in hospital or Morse Falls Assessment Scale >45; (4) moderate‐to‐severe cognitive impairment and communication difficulties; (5) severe co‐morbidity (stoke, cardiac instability); (6) missing data. A total of 56 patients who had experienced a postoperative fall in the hospital were enrolled as the observation group, and a control group of patients who had not experienced any falls was matched by gender. The study protocol was approved by the ethics committees of each centre, and the requirement for informed patient consent was waived due to the retrospective study design.

### 2.2 Data collection

- The Morse Falls Assessment Scale (Morse, 1986): This scale is a widely used fall risk assessment tool and was used in this study. The score ranges from 0 to 125 points, with higher scores representing a higher risk of falls. The scale traditionally includes six items: fall history (25 points), at least one co‐morbidity (15 points), mobile aids (15 or 30 points), intravenous tube or heparin pump (20 points), transfer or gait difficulties (10 or 20 points) and mental state (15 points). A higher final score indicates a higher fall risk, with a score >45. The Cronbach's α was 0.834, suggesting good reliability validity.
- Data collection: by consulting patient records, vascular surgery nurses collected relevant information, including gender, age, fall history before hospitalisation, lower limb fracture history before hospitalisation, continuously taking medication over 3 months, family support and at least one co‐morbidity (osteoporosis).

### 2.3 Statistical analysis

The SPSS version 26 software (IBM Corp.) was used to perform the statistical tests. A chi‐square or Fisher's exact test was used for the comparison of the categorical variables between the two groups. The independent risk factors for falls were analysed using multivariate logistic regression analysis (forward selection method). Differences were considered statistically significant when *p* values were <0.05.

## 3 RESULTS

The two groups comprised 56 patients who had experienced a fall as an observation group and 56 patients without falls as a control group. Observation group: 36 males and 20 females, aged 32–68 years (average 50.82 ± 8.14 years); body mass index (BMI) 18–30 kg/m^2^ (average 24.52 ± 2.74 kg/m^2^). Comparison group: 35 men and 21 women, aged 34–66 years (average 50.62 ± 8.64 years); BMI 19–30 kg/m^2^ (average 24.62 ± 2.67 kg/m^2^).

### 3.1 Univariate analysis of fall risk factors

As shown in Table 1, in the observed versus comparison groups, the percentage of males (64.29% vs. 62.50%) and the incidence of falls in the absence of family members (89.29% vs. 85.71%) were not statistically significant (*p* > 0.05). Compared with the comparison group, the observation group had a higher percentage of over 65 years old (71.43% vs. 37.50%), with a history of falls (69.64% vs. 21.43%), a history of fracture (62.50% vs. 17.86%), continuously took medication for more than 3 months (85.71% vs. 30.36%) and had a history of osteoporosis (58.93% vs. 8.93%). In terms of the frequency of falls, this was higher than in the comparison group, and the difference was statistically significant (all *p*s < 0.001).

**TABLE 1:** Univariate analysis of fall risk factors.

Factors	Observation group (*n* = 56)	Control group (*n* = 56)	*χ* ^2^	*p*
Sex				
Man	36 (64.29)	35 (62.50)	0.039	0.844
Woman	20 (35.71)	21 (37.50)		
Age				
>65 year old	40 (71.43)	21 (37.50)	12.997	<0.001
<65 year old	16 (28.57)	35 (62.50)		
A history of falls				
Yes	39 (69.64)	12 (21.43)	26.245	<0.001
No	17 (30.36)	44 (78.57)		
History of fracture				
Yes	35 (62.50)	10 (17.86)	23.217	<0.001
No	21 (37.50)	46 (82.14)		
Take medicine over 3 months				
Yes	48 (85.71)	17 (30.36)	35.231	<0.001
No	8 (14.29)	39 (69.64)		
Family members accompany				
Yes	50 (89.29)	48 (85.71)	0.327	0.568
No	6 (10.71)	8 (14.29)		
Osteoporosis				
Yes	33 (58.93)	5 (8.93)	31.226	<0.001
No	23 (41.07)	51 (91.07)		

### 3.2 Multivariate analysis of fall risk factors

Values from X1 to X5 included the factors that may contribute to a fall (age, fall history, fracture history, continuous medication over 3 months, osteoporosis) as independent variables (detailed in Table 2). Using multivariate logistic regression analysis, the following were included as independent risk factors for falls: age: 65 years old (OR: 1.334, 95% CI 1.021–1.496), a history of falls (OR: 1.324, 95% CI 1.021–1.385), a history of fracture (OR: 1.342, 95% CI 1.158–1.496), continuous medication for more than 3 months (OR: 1.339, 95% CI 1.254–1.402) and a history of osteoporosis (OR: 1.383, 95% CI 1.352–1.417). All of these differences were statistically significant (all *p*s < 0.05; Table 3).

**TABLE 2:** Assignments of fall risk factors.

Group	Hazards	Assignment
X1	Age	50 = 0, <50 = 1
X2	A history of falls	No = 0, Yes = 1
X3	History of fracture	No = 0, Yes =1
X4	Take medicine over 3 months	No = 0, Yes = 1
X5	Osteoporosis	No = 0, Yes = 1

**TABLE 3:** Multivariate analysis of fall risk factors.

Factor	*β*	Wald	*p*	OR	95% CI
Age	0.328	4.637	0.005	1.334	1.021–1.496
A history of falls	0.286	4.721	0.011	1.324	1.021–1.385
History of fracture	0.357	4.325	0.005	1.342	1.158–1.496
Take medicine over 3 months	0.357	4.024	0.012	1.339	1.254–1.402
Osteoporosis	0.395	4.134	0.021	1.383	1.352–1.417

## 4 DISCUSSION

Members of the elderly population include those at high risk of experiencing a fall. Most vascular surgery patients are older individuals with cognitive function decline, reduced activity endurance and mobility difficulties, making them prone to the risk of a fall (Jiang et al., 2021; Shi et al., 2020). According to previous studies, as age increases, the risk of falling will gradually increase (Chen et al., 2020; Yang et al., 2020). The occurrence of falls will not only increase the psychological burden of patients, but also cause a strong sense of physical discomfort, prolong the length of hospitalisation, increase the cost of treatment and affect a heavy burden on society and patients' families, all of which have attracted clinical attention (Shen et al., 2020).

This study showed that an age of 65 years, a history of falls and fractures, continuous medication for over 3 months and a history of osteoporosis were all risk factors for falls. This indicates that the occurrence of falls in vascular surgery patients is related to the following characteristics. (1) Old age: As people become older, skeletal muscle strength will decrease, particularly so in the quadriceps muscles, and long‐term sitting will cause muscle function degeneration and atrophy, thereby increasing the risk of falling. (2) A history of falls: In older patients who also have cataracts, vertigo, Parkinson's, cerebral thrombosis and other basic diseases, poor visual balance and peripheral neuropathy will greatly increase the risk of falling (Su et al., 2022). (3) A history of bone fracture: Patients with a history of fractures have weakened self‐care abilities, limb muscle strength and weakened activity endurance and are prone to adverse events and falls. In particular, patients with surgical complications such as bone non‐union have a higher risk of falling (Aso & Okamura, 2019; Zhou et al., 2021). (4) Taking medication for more than three consecutive months continuously. In combination with basic diseases, the older people will generally also use, for example calcium agents, vitamins, vascular expansion drugs, diuretics, antihypertensive drugs, anti‐anxiety and depression drugs, sedatives and hypnotic drugs. The application of these drugs can lead to adverse reactions such as reduced blood pressure, a weakened balance ability, gait imbalance, mental depression and confusion and ultimately increase the risk of falling (Bernet et al., 2022; Decalf et al., 2021). (5) A history of osteoporosis: Osteoporosis can lead to reduced muscle strength and impaired activity and elevates the risk of events such as falls. In particular, for patients who do not receive timely rehabilitative exercise, the risk of falling is higher (Glenn et al., 2021; Lin et al., 2020; Zhu & Sheng, 2020).

Because the movement functionality of the older people is weakened, the legs and feet are less flexible and activity speed slows down; additionally, when the older people are in a conversion position, they cannot move too fast, avoid the occurrence of postural hypotension and fall. For the characteristics of decreased stride and decline in the older people, a doctor should help them to strengthen their balance functionality, perform gait function checks, correctly analyse patient's gait. The pathological gait should be corrected in time, by strengthening the knee joint, advising on hip exercise, climbing stairs, walking, standing more muscle, muscle strength flexibility and enhance gait stability to reduce the risk of falls (Kiyoshi‐Teo et al., 2019; Tricco et al., 2021). More balance function training, standing, straightening the upper arms, hands clenched into fists, the upper body, upper arms twist to the left side and finally return to the original state and then twist to the right side and then return to the original state, so repeated training 5–10 times.

Most guidelines related to the risk of falls strongly recommended active management of fractures and osteoporosis as key elements in the prevention of falls. Osteoporosis is one of the important reasons for falls occurring among vascular surgery patients. Patients should be told to enhance their calcium intake and foods rich in vitamin D, such as soy products, nuts, seafood and milk. Clinical studies have shown that an adequate amount of vitamin D in daily diets can effectively prevent osteoporosis and reduce the risk of falling (Montero‐Odasso et al., 2021). Additionally, patients should be informed that the frequent use of hypoglycaemic drugs, antipsychotics, antidepressants and sedatives/sleeping drugs will increase the risk of falling. Patients should strictly follow their doctor's advice when taking drugs to ensure they ingest the correct quantity, on time and on demand and must not increase dosages by themselves to avoid adverse events such as falls (Montero‐Odasso et al., 2021).

Existing review findings have added new information showing that education has a positive effect on hospitalised fall rates and risk (Morris et al., 2019). Large, randomised trials showed the benefits of engaging patients and clinicians in education and training (Morris et al., 2019). When the patient is admitted to the hospital, the nurse should comprehensively and carefully introduce the ward environment to them and place usual items within easy reach of the patient. Patients with vertigo should be guided to sit down or use a bedpan. In conspicuous areas where a fall is likely to occur, carpets, wires and other potentially dangerous obstacles should be removed to avoid patients from tripping. Warning signs should be placed in all dangerous areas. In bathrooms, toilets, stairs, corridors and other areas where relevant, handrails should be set up; toilets must have handrails, instead of horizontal armrests. The patient's bed footbrake should be properly set, and the bed height must be appropriate; the ward environment must be well lit to ensure that patients have clear visibility (Hogan, Quigley, et al., 2021; Oshiro et al., 2019). In outdoor activities, patients should be accompanied by a family member and should walk with the help of a cane. Stairs must have handrails and patients should avoid walking unaccompanied.

This study has some limitations. First, it is a retrospective, small‐sample study. Although it included as many patients from five hospitals in Shanxi Province as possible, the results may still have been affected by selection bias, based on a small sample size. Second, this study only focused on inpatient fall events and did not conduct long‐term home‐based follow‐ups to explore the long‐term postoperative high risk factors related to falls. Future studies with large samples are needed to confirm the results of this study.

## 5 CONCLUSION

The occurrence of falls in vascular surgery patients is related to advanced age, a prior history of falls and fractures, continuous medication for more than 3 months and osteoporosis. There is a need for comprehensive falls assessments and patient education to mitigate fall risk in this vulnerable population.

## AUTHOR CONTRIBUTIONS

Yuan Bai and Xiao‐Hong Zhang conceived of the study, Fang He and Zhuo‐Xia Li participated in its design and coordination and Min Li and Ying Yu helped to draft the manuscript. All authors read and approved the final manuscript.

## FUNDING INFORMATION

This study was funded by the Shanxi Province ‘136 Revitalization Medical Project Construction Funds’.

## CONFLICT OF INTEREST STATEMENT

All of the authors had no personal, financial, commercial or academic conflicts of interest separately.

## ETHICS APPROVAL AND CONSENT TO PARTICIPATE

This study was conducted in accordance with the Declaration of Helsinki and approved by the Ethics Committee of Shanxi Bethune Hospital (ethical batch number: YXLL‐2022‐058). We obtained signed informed consent from the participants/legal guardians in this study.

## CONSENT FOR PUBLICATION

Not applicable.

## Data Availability

All data generated or analysed during this study are included in this published article.
